# Hepatitis B Virus Infection and Increased Risk of Gestational Diabetes Regardless of Liver Function Status: A Xiamen Area Population-Based Study

**DOI:** 10.3389/fphys.2022.938149

**Published:** 2022-07-11

**Authors:** Min Zhao, Shuyu Yang, Xiaojie Su, Tzu-Chieh Hung, Yishan Liu, Wenjie Zheng

**Affiliations:** ^1^ Department of Gynecology and Obstetrics, The First Affiliated Hospital of Xiamen University, School of Medicine, Xiamen University, Xiamen, China; ^2^ Computer Management Center, The First Affiliated Hospital of Xiamen University, School of Medicine, Xiamen University, Xiamen, China; ^3^ National Institute for Data Science in Health and Medicine, Xiamen University, Xiamen, China; ^4^ Research Studio of Traditional Chinese Medicine, The First Affiliated Hospital of Xiamen University, School of Medicine, Xiamen University, Xiamen, China; ^5^ Tongsheng High School, Linfen, China

**Keywords:** GDM, HBV, liver, retrospective, big data

## Abstract

**Background & Aims:** Hepatitis B virus (HBV) infection is a significant cause of liver function damage. However, previous studies on HBV mainly aimed at ordinary people, and there is a lack of consensus on the relationship between HBV infection and gestational diabetes mellitus (GDM) and whether HBV-infected pregnant women should undergo antiviral treatment. In addition, systematic studies on the impact of HBV infection on GDM have rarely been studied directly. Therefore, the overall goal of this study was to pursue the association between HBV infection, liver function, and GDM using Xiamen area gestational big data.

**Methods:** Using the Xiamen Primary Health Information System-maternal and child health information system, the data on participants (138,867 in total) expected confinement between 2008 and 2018 were included. Using univariate and multivariate logistic regressions, we constructed models to determine the role of HBV infection and liver function status in GDM. In addition, an analysis of variance tests was performed to study whether the relationship between HBsAg and GDM differed in the normal liver function and the abnormal liver function subgroups.

**Results:** HBsAg's positive status showed a substantial correlation with GDM onset in univariate and multivariate logistic regressions (*p* < 0.001). Subgroup analysis among HBsAg, liver function, and GDM suggests that both HBsAg and liver function affect the onset of GDM and have the highest prevalence of both abnormalities. Furthermore, ANOVA was used to investigate the association of HBsAg positive (*p* < 0.001), abnormal liver function (*p* < 0.001), and their interaction (*p* = 0.302) on the onset of GDM. This result showed that HBsAg is an independent factor of GDM pathogenesis, regardless of liver function status.

**Conclusion:** HBsAg and liver function are independent factors in GDM. Therefore, regarding these results, while clinicians consider the traditional risk factors of GDM, it is necessary to consider the HBV infection status. Conducting a dietary intervention for HBsAg-positive pregnant women at the early stage of pregnancy is conducive to reducing the adverse effects.

## 1 Introduction

Gestational diabetes mellitus (GDM) was defined as any degree of glucose intolerance that was first recognized during pregnancy, regardless of hyperglycemia (Zhang et al., 2010). The incidence of GDM has shown an upward trend recently, approaching the incidence of type 2 diabetes and obesity. According to reports, the incidence of gestational diabetes is about 16–18% (Musilova et al., 2012) and about 7.7% in China (Atlas, 2015).

The pathogenesis of GDM is not entirely understood. From an evolutionary point of view, placental hormone secretion antagonizes insulin action in the medium and later term of pregnancy, which leads to physiological increase in insulin resistance. This mechanism is designed to limit maternal glucose utilization and thereby shunt an adequate amount of supply to the growing fetus (Hay, 2006). When maternal insulin secretion cannot compensate for pregnancy-induced insulin resistance, it leads to maternal hyperglycemia, which causes severe complications of GDM and obesity (Kampmann et al., 2019). GDM carries risks for the mother, fetus, and neonate. The Hyperglycemia and Adverse Pregnancy Outcome (HAPO) study demonstrated that the risk of adverse maternal, fetal, and neonatal outcomes continuously increased as a function of maternal glycemia at 24–28 weeks of gestation, even within ranges previously considered normal for pregnancy (Metzger et al., 2008). Therefore, early diagnosis of gestational diabetes and lifestyle interventions for high-risk pregnant women can reduce the risk of gestational diabetes and reduce adverse outcomes. However, the current clinical diagnosis of gestational diabetes is mainly based on oral glucose tolerance tests at 24–28 weeks of pregnancy, which brings difficulties to early diagnosis and highlights the importance of screening and close follow-up in the high-risk group such as older or obese pregnant women.

Hepatitis B virus (HBV) infection is one of the most prevalent infectious diseases, affecting about two billion people worldwide, among which about 5% of pregnant women have chronic HBV infection (Li et al., 2011). HBV infection is a routine screening program for pregnant women, with a view to early detection of pregnant women with abnormal liver function caused by HBV replication or pregnant women with high virus load prone to mother-to-child transmission. HBV infection is associated with multiple pregnancy complications, such as spontaneous preterm birth and habitual abortion (Atalay et al., 2016). In addition, previous studies have also reported that HBV could continuously destroy islet cells, causing insulin secretion deficiency to induce GDM (Shetty et al., 2000; Kim et al., 2012; Rawat and Boucharf, 2015; Barthel et al., 2016). A study has shown that the incidence of diabetes in patients with chronic liver disease is significantly higher than that in the healthy population (Rouabhia et al., 2010). However, the association between HBV infection and GDM remains inconclusive, especially in HBV-infected pregnant women with a normal liver function who were often overlooked in clinical practice.

HBV infection is a significant cause of liver function damage. The liver is the principal organ to secrete bile and store glycogen and vital in maintaining blood sugar stability. Changes in maternal hormone levels and the immune status during pregnancy can affect HBV the infection status, affect liver function, aggravate glucose metabolism disorders, and affect the course of pregnancy. However, previous studies on HBV are primarily aimed at ordinary people, and there is a lack of consensus on the relationship between HBV infection and GDM and whether HBV-infected pregnant women should undergo antiviral treatment. Although the vertical transmission of HBV from the mother to the child has been effectively controlled with the widespread use of the hepatitis B vaccine, systematic studies on the impact of HBV infection on GDM have rarely been studied directly. Therefore, the overall goal of this study was to pursue the association between HBV infection, liver function, and GDM using Xiamen area gestational big data.

## 2 Methods

### 2.1 Patient Selection

This retrospective cohort study was conducted under the ethical guidelines of the Declaration of Helsinki and was approved by the review board of the First Affiliated Hospital of Xiamen University. The participants whose expected date of confinement was between 2008 and 2018 were collected in this study using the area population-based clinical data from the Xiamen Primary Health Information System-maternal and child health information system. This system is a governmental data warehouse, established and managed by the Xiamen Municipal Health Committee. It consisted of the public life-cycle medical information collected from 28 primary medical institutions in Xiamen, including birth, adolescence, childbearing, menopause, and old age. The inclusion criteria accepted in the present study were as follows: 1) aged below 40 years old; 2) BMI under 28; and 3) no history of a previous diabetes diagnosis. The exclusion criteria were as follows: the person was unconscious or had critically ill, learning difficulties, or severe mental illness, and the fetus had an abnormal ultrasound result.

### 2.2 Diagnosis Standard

The GDM diagnostic criteria used in this study were the International Association of Diabetes and Pregnancy Study Group (IADPSG) standard. Pre-gestational diabetes mellitus (PGDM) patients are excluded before the 75 g OGTT. The fasting plasma glucose level, 1-h, and 2-h blood glucose levels after the oral glucose admission were measured. The typical blood glucose values should be less than 5.1 mmol/L, 10.0 mmol/L, and 8.5 mmol/L (92, 180, and 153 mg/dl). A diagnosis of GDM occurs when any blood glucose level meets or exceeds the criteria.

The HBsAg status inclusion criteria for this study were as follows: maternal HBV infection (prenatal screening HBsAg positive). The normal interval of the liver function indicators is defined as follows: ALT [5, 40] U/L, AST [0, 40] U/L, ALB [35, 50] g/L, TBil [3.4, 20.5] μmol/L, and DBil [0, 6.84] umol/L.

### 2.3 Clinical Variables

The following clinical variables of patients were included and analyzed: alcohol, cardiopathy, pneumopathy, hypertension, smoking, epilepsy, hyperthyroidism, nephropathy, hematopathy, family diabetes history, folic acid consumption, menarche age, hemoglobin (HBG), white blood count (WBC), platelet count (PLT), serum creatinine (SCr), blood urea nitrogen (BUN), body mass index (BMI), age, alanine aminotransferase (ALT), aspartate aminotransferase (AST), albumin (ALB), total bilirubin (TBil), direct bilirubin (DBil), international normalized ratio (INR), and hepatitis B surface antigen (HBsAg). In addition, according to the liver function test, the patients were further classified into liver function normal and abnormal cohorts to investigate the association between the HBV status, liver function status, and diagnosis of GDM. The definition of the abnormal liver function status accepted in the present study was defined as the patients who harbored any abnormal liver function indicators, such as liver enzymes, bilirubin, albumin, and INR.

### 2.4 Data Processing and Statistical Analysis

**FIGURE 1:**
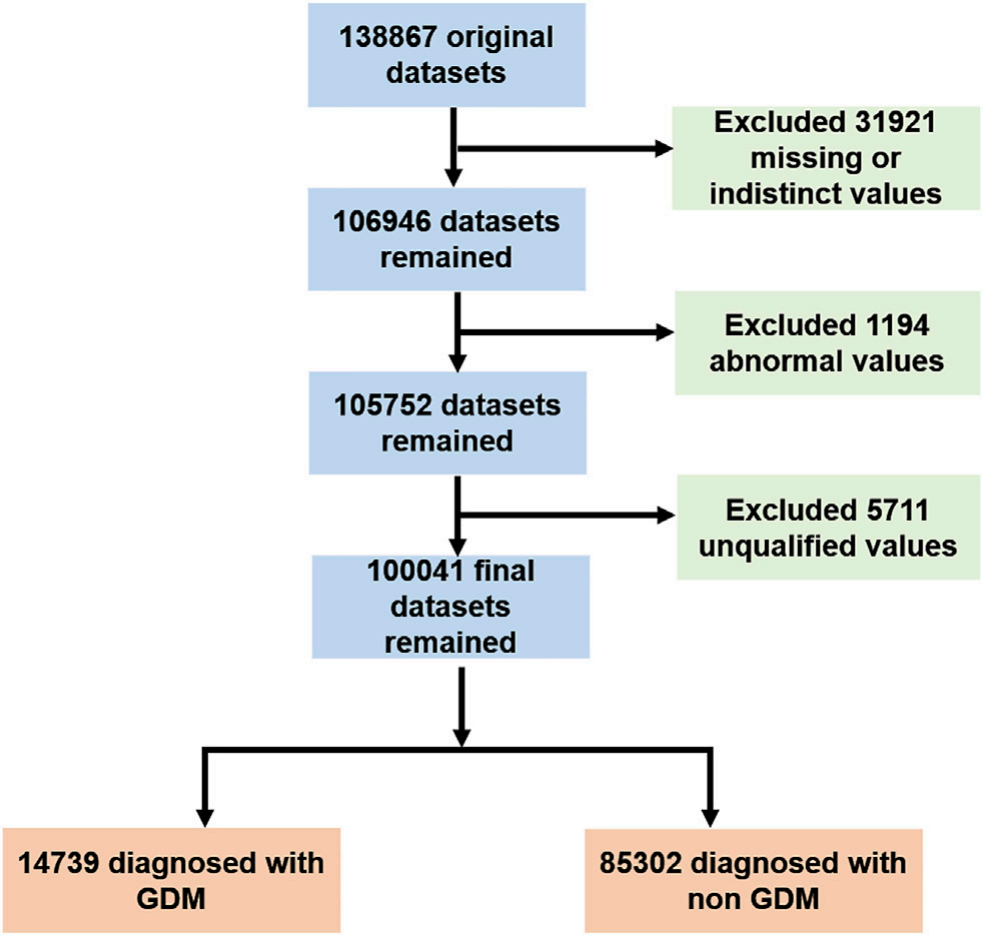
Selection of the study population.

An extract-transform-load process was performed through SQL server 2008 R2 database software (Microsoft), and the patients’ data containing missing indistinct values were excluded. As a result, 0.25% of extreme data at both ends were truncated; thus, 99.5% of the remaining data were investigated (Figure 1).

Continuous data were expressed as the mean and standard deviation (SD) and were analyzed using t-tests. Categorical data were presented as count and percentage and were compared using Pearson’s chi-squared tests. Univariate and multivariate logistic regressions were applied to determine the role of HBV infection and liver function status in GDM. In order to further study whether the relationship between HBsAg and GDM was different in the normal liver function and the abnormal liver function subgroups, analysis of variance (ANOVA) tests were performed.

All statistical tests were two-sided, made at a 5% significance level, and performed using R version 4.1.1 (R Project, Vienna, Austria).

## 3 Results

### 3.1 Baseline Characteristics

A total of 100,041 pregnant women who met the inclusion and exclusion criteria were included in the present study (as shown in Figure 1). The baseline characteristics of the total cohort and the comparison of clinical variables between HBsAg positive and negative cohorts are shown in Table 1. The result showed that the positive HBsAg cohort was significantly associated with a high proportion of GDM (*p* < 0.001), while the other variables were not statistically significant between these two cohorts.

**TABLE 1:** Baseline characteristics in HBsAg-negative and -positive groups.

Variable	HBsAg-positive (*n* = 10,355)	HBsAg-negative (*n* = 89,686)	*p*-value
**Alcohol +**, N (%)	3 (0.03%)	42 (0.05%)	0.571
**Cardiopathy +**, N (%)	22 (0.21%)	214 (0.24%)	0.680
**Pneumopathy +**, N (%)	15 (0.14%)	173 (0.19%)	0.343
**Hypertension +**, N (%)	15 (0.14%)	118 (0.13%)	0.835
**Smoking +**, N (%)	2 (0.02%)	19 (0.02%)	1
**Epilepsy +**, N (%)	4 (0.04%)	37 (0.04%)	1
**Hyperthyroidism +**, N (%)	96 (0.93%)	802 (0.89%)	0.779
**Nephropathy +**, N (%)	34 (0.33%)	331 (0.37%)	0.572
**Hematopathy +**, N (%)	29 (0.28%)	174 (0.19%)	0.084
**Family diabetes history +**, N (%)	588 (5.68%)	4,908 (5.47%)	0.396
**Folic acid consumption +**, N (%)	10,167 (98.18%)	88,028 (98.15%)	0.843
**Menarche**, mean (SD), year	14.17 (1.43)	14.15 (1.43)	0.115
**Hemoglobin**, mean (SD), g/l	123.61 (10.18)	123.64 (10.18)	0.841
**White blood count**, mean (SD), 10^9^/L	8.34 (1.95)	8.35 (1.94)	0.314
**Platelet count**, mean (SD), 10^9^/L	229.40 (51.21)	229.21 (50.75)	0.583
**Serum creatinine**, mean (SD), mol/L	55.01 (13.88)	54.95 (13.69)	0.981
**Blood urea nitrogen**, mean (SD), mmol/L	2.80 (1.14)	2.82 (1.19)	0.138
**Body mass index**, mean (SD)	20.35 (2.42)	20.36 (2.43)	0.480
**Age,** mean (SD), year	28.07 (4.16)	28.10 (4.16)	0.322
**Alanine aminotransferase**, mean (SD), U/L	17.16 (12.23)	17.22 (12.20)	0.230
**Aspartate aminotransferase**, mean (SD), U/L	17.73 (6.70)	17.80 (6.76)	0.554
**Albumin**, mean (SD), g/L	42.29 (4.01)	42.29 (3.99)	0.916
**Total bilirubin**, mean (SD), μmol/L	10.61 (4.70)	10.57 (4.63)	0.484
**Direct bilirubin**, mean (SD), μmol/L	3.63 (2.62)	3.61 (2.55)	0.546
**GDM +**, N (%)	1,691 (16.33%)	13,048 (14.55%)	<0.001

### 3.2 Association Between Hepatitis B Virus Infection and Gestational Diabetes

As shown in Table 2, univariate logistic regression analysis identified that 15 variables were associated with GDM diagnosis (both *p* < 0.05), among which the HBsAg positive status was positively associated with GDM (odds ratio [OR] with 95% confidence interval [CI] = 1.147 [1.086–1.212]; *p* < 0.001). The further multivariate logistic analysis identified that the HBsAg status remained as the independent risk factor for GDM after the correction of other confound variables (odds ratio [OR] with 95% confidence interval [CI] = 1.174 [1.167–1.180]; *p* < 0.001). In addition to the significant correlation between the HBsAg status and GDM onset, the result of the present study was that several liver function indicators, such as high levels of AST, ALT, and TBIL, showed a substantial correlation with GDM onset in both uni- and multivariate analyses as well.

**TABLE 2:** Univariate and multivariate logistic regressions in GDM populations.

Variable	Univariate logistic regression				Multivariate logistic regression			
	Estimate	OR	95% CI	*p*-value	Estimate	OR	95% CI	*p*-value
Alcohol +	−0.116	0.890	(0.377, 2.105)	0.791	−0.189	0.828	(0.337, 2.031)	0.680
Cardiopathy +	0.226	1.254	(0.898, 1.749)	0.185	0.083	1.087	(0.762, 1.549)	0.648
Pneumopathy +	0.013	1.013	(0.678,1.514)	0.950	−0.009	0.991	(0.649, 1.513)	0.967
Hypertension +	0.804	2.234	(1.528, 3.268)	<0.001	0.137	1.147	(0.760, 1.731)	0.514
Smoking +	−1.240	0.289	(0.040, 2.120)	0.222	−0.825	0.438	(0.056, 3.411)	0.431
Epilepsy +	−0.008	0.992	(0.417, 2.359)	0.986	0.092	1.096	(0.441, 2.728)	0.843
Hyperthyroidism +	0.076	1.079	(0.901, 1.292)	0.411	−0.102	0.903	(0.747, 1.092)	0.296
Nephropathy +	−0.039	0.962	(0.717, 1.290)	0.793	−0.002	0.998	(0.732, 1.360)	0.991
Hematopathy +	−0.077	0.926	(0.621, 1.381)	0.705	−0.027	0.973	(0.639, 1.483)	0.900
Family diabetes history +	0.220	1.246	(1.159, 1.340)	<0.001	−0.023	0.977	(0.905, 1.055)	0.557
Folic acid consumption +	0.207	1.230	(1.070, 1.414)	0.004	0.059	1.061	(0.914, 1.231)	0.439
Menarche (age)	−0.015	0.985	(0.974, 0.997)	0.016	−0.010	0.990	(0.977, 1.004)	0.146
Hemoglobin/HBG (g/L)	0.018	1.018	(1.016, 1.020)	<0.001	0.009	1.009	(1.007, 1.011)	<0.001
White blood count/WBC (/μL)	0.105	1.111	(1.102, 1.119)	<0.001	0.075	1.078	(1.067, 1.088)	<0.001
Platelet count/PLT (*10^9^/L)	0.003	1.003	(1.001, 1.005)	<0.001	0.001	1.001	(0.999, 1.003)	0.002
Serum creatinine/SCr (mol/L)	−0.001	0.999	(0.997, 1.001)	0.269	−0.003	0.997	(0.995, 0.999)	<0.001
Blood urea nitrogen/BUN (mmol/L)	0.015	1.015	(1.001, 1.029)	0.036	−0.013	0.987	(0.970, 1.005)	0.130
Body mass index/BMI	0.233	1.262	(1.253, 1.272)	<0.001	0.178	1.195	(1.185, 1.204)	<0.001
Alanine aminotransferase/ALT(U/L)	0.013	1.013	(1.011, 1.015)	<0.001	0.012	1.012	(1.010, 1.014)	<0.001
Aspartate aminotransferase/AST(U/L)	0.010	1.010	(1.008, 1.012)	<0.001	−0.006	0.994	(0.990, 0.998)	0.001
Albumin/ALB (g/L)	−0.001	0.999	(0.995, 1.003)	0.772	0.008	1.008	(1.002, 1.014)	0.002
Total bilirubin/TBil (μmol/L)	−0.011	0.989	(0.985, 0.993)	<0.001	−0.006	0.994	(0.990, 0.998)	0.008
Direct bilirubin/DBil (μmol/L)	−0.019	0.981	(0.974, 0.989)	<0.001	−0.009	0.991	(0.983, 0.999)	0.055
HBsAg	0.137	1.147	(1.086, 1.212)	<0.001	0.160	1.174	(1.167, 1.180)	<0.001
Age	0.164	1.178	(1.174, 1.183)	<0.001	0.150	1.162	(1.157, 1.166)	<0.001

### 3.3 Subgroup Analysis of the Liver Function Status

We further analyzed the data into two groups to investigate the relationship between the prevalence of GDM and HBV infection in the normal liver function and abnormal liver function groups.

The results showed a positive correlation between HBsAg and GDM, regardless of liver function. In the group with abnormal liver function, the prevalence of GDM in HBsAg-positive pregnant women was 18.38%; for HBsAg-negative pregnant women, it was 15.78%. In the group with normal liver function, the prevalence of GDM in HBsAg-positive pregnant women was 15.90%, and for HBsAg negative pregnant women, it was 14.30% (Table 3).

**TABLE 3:** GDM prevalence in different groups.

GDM prevalence	Liver function (abnormal)	Liver function (normal)
H (%)BsAg +	18.38	15.90
HBsAg -	15.78	14.30

**FIGURE 2:**
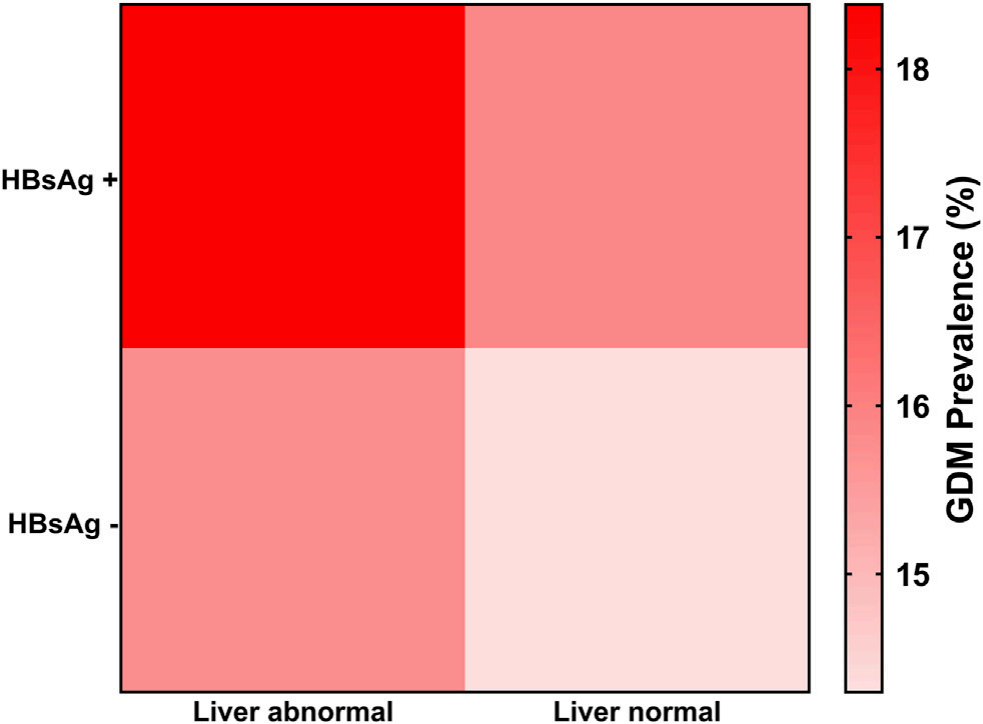
GDM prevalence in live function normal and abnormal groups.

Interestingly, the prevalence of GDM showed a step-by-step increase with the liver function status and HBsAg status. The prevalence ranged from 14.30 to 18.38% (Figure 2). This result suggests that both HBsAg and the liver function affect the onset of GDM and have the highest prevalence of both abnormalities.

### 3.4 Variance Analysis

Furthermore, ANOVA was used to investigate the association of HBsAg-positive status (*p* < 0.001), abnormal liver function (*p* < 0.001), and their interaction with the onset of GDM. The results showed that both factors were statistically significantly associated with GDM onset. However, the combined interaction (*p* = 0.302) was not statistically significant with GDM onset (Table 4). It shows that abnormal liver function and HBsAg-positive status are independent factors of GDM.

**TABLE 4:** Variance analysis of HBsAg, liver function, and their interaction on the onset of GDM.

Factor	*p*-value
HBsAg status	<0.001
Liver function	<0.001
Interaction of HBsAg and liver function	0.302

## 4 Discussion

Our previous study (Zhao et al., 2021) identified eight independent pre-/early-pregnancy predictors, namely, pre-pregnancy BMI, pre-pregnancy intake of folic acid, white cell count, platelet count, alanine transaminase, albumin, direct bilirubin, and creatinine, which were significantly associated with the later GDM risk. Moreover, we established a nomogram model with these predictors, which had an over-roll of 85% accuracy in early detection of GDM progress based on the pregnant women’s clinical data from the Xiamen Primary Health Information System-maternal and child health information system.

### 4.1 Liver Function and Gestational Diabetes Mellitus

Our results showed a statistically significant association of liver function indicators with GDM disease in the present study. ALT and AST are the primary indicators of the evaluation of liver function. The results showed that ALT and AST in the GDM group were significantly higher than in the non-GDM group. HBV virus infection carries liver injury as the primary clinical symptom. In glycogenolysis, glucose polymers are stored in the liver glycogen. In glycolysis, glycogen can be decomposed into glucose and converted into energy. The synthesis and degradation of liver glycogen are controlled by insulin. Under normal conditions, the body can synthesize and degrade liver glycogen. HBV virus infection can cause hepatocyte necrosis and inflammatory cell infiltration. This phenomenon is pronounced under high viral load HBV infection (active virus period)–induced liver damage; liver glycogen synthesis damage can cause elevated blood sugar levels. Some scholars believe that autoimmune pancreatitis caused by high load HBV virus infection leads to autoantibodies against insulin cells and is the critical factor of GDM (Spardling et al., 2013; Sarkar and NorahTerrault, 2014). Bilirubin acts as a biochemical antioxidant to inhibit radical oxygen formation and lipid oxidation, and it also inhibits inflammatory and immune responses (Mark, 2013). HBV may be interfering with glucose homeostasis through insulin sensitivity. This effect may have bypassed the protective mechanism of the antioxidants. Barthel’s result shows that HBV induces activation of nuclear factor erythroid 2 related factors 2 (Nrf2), which causes intracellular retention of the Insulin receptor (INSR), reducing cell surface functional INSR, further induces the weakening of insulin binding, and causes insulin signaling inhibition (Barthel et al., 2016). Kim’s research demonstrated that the six trans-membrane protein of prostate 2 (STAMP2) protein physically interacts with and decreases the hepatitis B virus X protein (HBx) stability, thereby counteracting HBx-induced hepatic lipid accumulation and insulin resistance (Kim et al., 2012). In addition, recent studies have found that HBX interferes with the host cell PI3K/Akt insulin signaling pathway, leading to impaired insulin signaling transduction and a decline in liver cell glycogen synthesis, and elevated blood glucose concentration (Rawat and Boucharf, 2015).

### 4.2 HBsAg and Gestational Diabetes Mellitus

Our results showed that HBsAg was an independent factor in GDM. HBV-induced liver injury is usually accompanied by different degrees of pancreas injury. Some basic tissue embryology research (Shetty et al., 2000) shows that the liver and pancreas have the exact embryonic origin and similar tissue structures. HBV virus also has a solid affinity for pancreatic tissue. HBV virus particles can be found in pancreatic biopsy in some patients with cirrhosis. Studies have shown that (Sirilert et al., 2014) patients with severe HBV infection and cirrhosis can have increased insulin resistance, and some patients can have abnormal glucose metabolism. Pregnant women with HBV virus infection can lead to islet beta-cell injury, and the decrease of insulin secretion directly induces gestational diabetes mellitus.

### 4.3 Liver Function and Hepatitis B Virus Interaction on Gestational Diabetes Mellitus

Our results further suggest that HBsAg is an independent factor of GDM pathogenesis, regardless of the liver function status. Liver function and HBV interaction did not have a statistical significance with GDM.

### 4.4 Medical Nutrition Therapy and Gestational Diabetes Mellitus

Our result demonstrates that pregnant women infected by HBV, regardless of their liver function status, are more vulnerable to GDM. Therefore, we recommend that clinicians and policymakers consider health management strategies for HBV-infected pregnant women at the early stage of their pregnancy. MNT has been widely used in clinical practice (Erkamp et al., 2020; Liu et al., 2020). MNT determines the medical and nutritional treatment program through high-risk screening of pregnant women and changes unhealthy lifestyles through nutrition education during pregnancy, individualized blood sugar levels, and weight management. The content of the MNT protocol includes diet and physical exercise, and it is a clinical non-pharmacological therapeutic intervention for pregnant women with GDM. Badon’s study has suggested the management of an unhealthy lifestyle in early pregnancy, including diet, physical exercise, smoking cessation, and stress management. This study believes that earlier health management of blood sugar, blood fats, and body weight during pregnancy can reduce the risk of pregnancy-associated syndromes such as GDM (Badon et al., 2018). In addition, MNT programs, including dietary and exercise intervention protocols, are beneficial in reducing the risk of complications of GDM from the glucose control disorder during pregnancy (Cosson et al., 2017).

### 4.5 Limitation

A significant limitation is that the hepatitis B vaccine–inoculated groups were not excluded in this study, requiring further investigation. In terms of current practice, the present study is a local, regional study using IADPSG criteria to define GDM, which, although recommended by the World Health Organization (WHO, 2013), is not universally adopted (NICE, 2015). Glycohemoglobin is a critical reference parameter for diagnosing gestational diabetes mellitus (Powe, 2017); this study’s lack of glycohemoglobin data is also a deficiency.

## 5 Conclusion

In conclusion, this study demonstrates an association between HBsAg and an increased risk of gestational diabetes mellitus, regardless of the liver function status, which may have implications for the clinical diagnosis and treatment of GDM: intensive follow-up for GDM should be required for HBV-infected patients with or without liver function damage. Regarding these results, while clinicians consider the traditional risk factors of GDM, it is necessary to consider the HBV infection status. Conducting an MNT or undergoing antiviral treatment for HBsAg-positive pregnant women at the early stage of pregnancy is conducive to reducing the adverse effects.

## Data Availability

The original contributions presented in the study are included in the article/Supplementary Material; further inquiries can be directed to the corresponding author.
